# MR Spectroscopy in Patients with Hereditary Diffuse Leukoencephalopathy with Spheroids and Asymptomatic Carriers of Colony-stimulating Factor 1 Receptor Mutation

**DOI:** 10.2463/mrms.mp.2016-0016

**Published:** 2016-12-26

**Authors:** Takashi Abe, Toshitaka Kawarai, Koji Fujita, Wataru Sako, Yuka Terasawa, Tsuyoshi Matsuda, Waka Sakai, Ai Tsukamoto-Miyashiro, Naoko Matsui, Yuishin Izumi, Ryuji Kaji, Masafumi Harada

**Affiliations:** 1 Departments of Radiology, Institute of Biomedical Sciences, Tokushima University Graduate School, 2-50-1 Kuramoto-cho, Tokushima, Tokushima 770-8509, Japan; 2 Clinical Neuroscience, Institute of Biomedical Sciences, Tokushima University Graduate School, Tokushima, Japan; 3 Department of Neurology, Jikei University School of Medicine, Tokyo, Japan; 4 MR Applications and Workflow Asia Pacific, GE Healthcare Japan Corporation, Tokyo, Japan

**Keywords:** magnetic resonance imaging, hereditary diffuse leukoencephalopathy with spheroids, colony stimulating factor-1 receptor, Mescher-Garwood point-resolved spectroscopy

## Abstract

**Purpose:**

Hereditary diffuse leukoencephalopathy with spheroids (HDLS) is a rare neurodegenerative disorder with various clinical presentations. Mutation of the colony-stimulating factor 1 receptor (*CSF1R*) gene is considered to be a cause of this autosomal dominant disorder. The purpose of this study was to report magnetic resonance spectroscopy (MRS) findings in patients with HDLS and asymptomatic carriers and to clarify the use of MRS in this disease.

**Materials and Methods:**

In this retrospective, institutional review board-approved study, we included four consecutive patients, genetically diagnosed with HDLS, and two asymptomatic carriers after acquiring informed consent. We performed single-voxel MRS of the left centrum semiovale on a 3T clinical scanner. We also included a sex-matched normal dataset. We quantified N-acetylaspartate (NAA), creatine, choline-containing compounds (Cho), glutamine, glutamate (Glu), myo-inositol (Ins), glutathione, lactate (Lac), and gamma-amino butyric acid using LCModel. We performed statistical analysis, and *P* value <0.05 was considered significant.

**Results:**

In HDLS cases, MRS revealed decreased NAA and Glu concentrations, which probably reflected neuronal damage and/or loss, and a subsequent reduction of neurotransmitters. A patient with HDLS also had increased Cho and Ins concentrations, indicating gliosis, and increased Cho concentration was also observed in an asymptomatic carrier. This suggests that metabolic changes had already occurred in an asymptomatic state.

**Conclusion:**

We demonstrated changes in metabolite concentrations not only in patients with HDLS but also in asymptomatic *CSF1R* mutation carriers. Our study indicates that MRS is a potentially useful tool for the analysis of metabolic and pathophysiological findings of HDLS, even during the early stages of disease.

## Introduction

Hereditary diffuse leukoencephalopathy with spheroids (HDLS) is a rare neurodegenerative disorder with autosomal dominant inheritance.^1,2^ The main symptoms are personality change, dementia, parkinsonism, seizures and depression.^3^ The age at onset is variable, from 8 to 78 years and this disease gradually progresses. Previously, histological analysis was required for a definitive diagnosis; however, in 2011, Rademakers et al. reported a disease-specific genetic mutation affecting the tyrosine kinase domain of colony-stimulating factor 1 receptor (*CSF1R*).^4^ Researchers subsequently published many reports on clinical and genetic diagnoses of HDLS.^3,5–7^

Diagnostic imaging, including magnetic resonance imaging (MRI), reveals the characteristic findings in patients with HDLS.^8,9^ Cerebral atrophy, especially in the frontal and parietal lobes, and white matter hyperintensity on T_2_-weighted imaging (T_2_WI), fluid-attenuated inversion recovery (FLAIR), and diffusion-weighted imaging (DWI) are frequent findings. Corpus callosal atrophy is a hallmark of this disease.^10^ White matter lesions extend through the corticospinal tract from the cerebrum to the brainstem and spinal tract.^11^ Calcification may occur in the cerebral gray and white matter.^12^

Magnetic resonance spectroscopy (MRS) can detect changes in metabolites in the brains of patients with various diseases. Previous reports described decreased N-acetylaspartate (NAA) concentration, and increased choline-containing compounds (Cho) and myo-inositol (Ins) concentrations in patients with HDLS, with gradual exacerbation of these abnormal findings.^13–15^ However, reports of HDLS-specific MRS findings are limited, and the change in glutamine (Gln), glutamate (Glu), glutathione (GSH), and gamma-amino butyric acid (GABA) concentrations in affected patients have not yet been reported.

Recently, Riku et al. reported pathological findings in an asymptomatic carrier of the causative gene, *CSF1R*; this patient did not present any clinical presentations of HDLS at that time but exhibited the same pathological features as those seen in a patient with HDLS.^16^ In addition, some reports described abnormal conventional brain MRI findings in carriers even before HDLS development.^3,17^ Brain metabolism might be altered in asymptomatic carriers; however, to our knowledge, no MRS alteration has been reported in this population.^18^

The purpose of this study was to report MRS findings in patients with HDLS and asymptomatic carriers and to clarify the use of MRS in this disease.

## Materials and Methods

### Subjects

**Fig 1.**
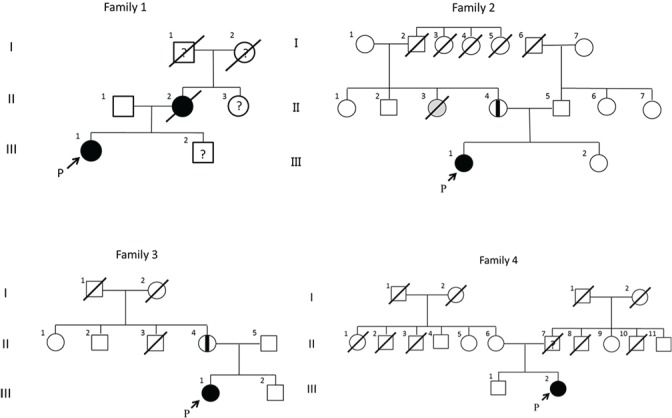
Pedigree charts. Unknown phenotype is indicated by a question mark. Mild cognitive impairment is indicated by a filled gray symbol. Asymptomatic carriers are represented by a line down the middle of the symbol. Solid symbols, affected individuals; circles, female subjects; squares, male subjects; slashes, deceased; arrow, proband.

In this retrospective, institutional review-board approved study, we included four consecutive patients, diagnosed with HDLS by experienced neurologists and confirmed to possess a genetic abnormality in *CSF1R*. After we explained the disease and its hereditary nature to the patients’ families, we also performed genetic sequencing on samples from family members who provided consent. Two were diagnosed as asymptomatic carriers and participated in this study after providing informed consent (Fig. 1). We used in-house MRI and MRS datasets obtained from healthy female subjects for comparison. We searched studies performed from April 2012 to March 2015 and identified 13 short TE MRS and five MEGA-PRESS datasets obtained from the left centrum semiovale (CS) (mean age: 59 years, range: 33–80). Table 1 summarizes the characteristics of these patients, carriers and normal datasets.

### Imaging protocol

**Fig 2.**
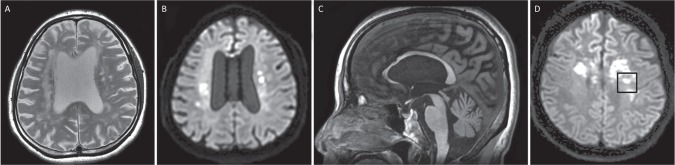
Magnetic resonance imaging of a patient with hereditary diffuse leukoencephalopathy with spheroids (Family 4. III-2) reveals white matter hyperintensity on T_2_-weighted imaging (**A**) and diffusion-weighted imaging (**B**) along with brain atrophy, particularly in the corpus callosum (**C**) and the frontal and parietal lobes. The volume of interest for short TE magnetic resonance spectroscopy (**D**, square) includes hyperintensity on diffusion-weighted imaging.

**Fig 3.**
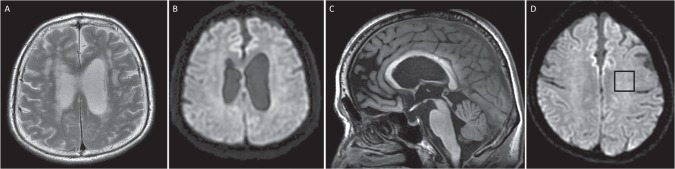
Magnetic resonance imaging of an asymptomatic carrier (Family 3. II-4) shows white matter hyperintensity on T_2_-weighted imaging (**A**), with brain atrophy particularly in the frontal and parietal lobes; however, the hyperintense areas are smaller and atrophy is weaker than that observed in a patient with HDLS (Fig. 1). Diffusion-weighted imaging does not reveal abnormal hyperintensity (**B**) or corpus callosal atrophy (**C**). The volume of interest for short TE magnetic resonance spectroscopy (**D**, square) doesn’t include abnormal intensity on diffusion-weighted imaging. HDLS, Hereditary diffuse leukoencephalopathy with spheroids.

We used a 3T scanner with an 8-channel head coil (Discovery 750; GE Healthcare, Milwaukee, WI, USA). We conducted T_1_-weighted imaging (T_1_WI, repetition time/echo time, TR/TE, 520/12 msec; field of view, FOV 240 mm; section thickness, 6 mm; matrix, 384 × 256), T_2_WI (TR/TE 6000/96 msec; FOV 240 mm; section thickness, 6 mm; matrix, 512 × 320), FLAIR (TR/TE/inversion time, 12000/140/2200 msec; FOV, 240 mm; section thickness, 6 mm; matrix, 320 × 192), and DWI (TR/TE, 4400/61 msec; b-factor, 1000 sec/mm^2^; FOV, 240 mm; section thickness, 6 mm; matrix, 160 × 256). Imaging findings are summarized in Table 1. Figs. 2 and 3 are representative images of a patient with HDLS and an asymptomatic carrier, respectively.

**Fig 4.**
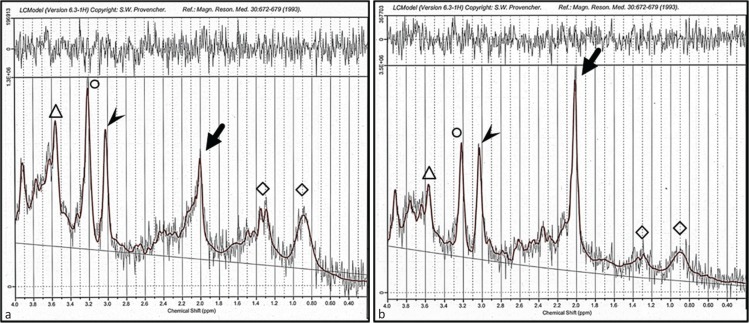
Short TE magnetic resonance spectroscopy (MRS) of a patient with hereditary diffuse leukoencephalopathy with spheroids (**a**, Family 4. III-2) shows a decrease of NAA (arrow) and increase of Cho and Ins (circle and triangle, respectively). Increase of Lac and/or lipid were also suspected in 1.3 and 0.9 ppm (square). MRS of an asymptomatic carrier (**b**, Family 3. II-4) is almost normal except for a slight increase of Cho, and accumulation of Lac and/or lipid are suspected. Arrowheads indicate the concentration of Cr. NAA, N-acetyl aspartate; Cho, choline-containing compounds; Ins, myo-inositol; Lac, lactate; Cr, creatine and phosphocreatine.

We performed short TE single-voxel MRS with stimulated echo acquisition mode (STEAM) and Mesher–Garwood point resolved spectroscopy (MEGA-PRESS) of the left CS. The scan parameters were as follows: STEAM: volume of interest (VOI), 15 × 20 × 20 mm; TR, 5000 ms; TE, 15 ms; number of excitations, 48; acquisition time, 5:40 min; MEGA-PRESS: VOI, 30 × 30 × 30 mm; TR, 1500 ms; TE 68 ms; number of excitations, 256; acquisition time, 6:54 min. One patient with HDLS was excluded from the study because neuroimaging data was not available (Family 4, III-2). Figures 2D and 3D are examples of VOI setting for short TE MRS. VOI for the cases with HDLS included abnormal hyperintensity on DWI, but VOI for the asymptomatic carriers didn’t (Figs. 2D and 3D). Figure 4 represented examples of short TE MRS spectra.

### Quantification of each metabolite

We quantified each metabolite using LCModel, the software reported by Provencher et al.^19^ (Version 6.3-1K) with an in-house basis-set. We used an unsuppressed water signal as an internal reference for metabolite quantification. We assumed concentration of water in the brain as 35880 mM.

We quantified NAA, creatine (Cre), Cho, glutamine–glutamate complex (sum of Gln and Glu [Glx]), Gln, Glu, Ins, GSH, and lactate (Lac) from short TE MRS data. We acquired GABA concentration from MEGA-PRESS data. We used the data with Cramer–Rao lower bound below 30%.

### Statistical analysis

We compared differences in age and metabolite concentrations among patients with HDLS, carriers and normal datasets. Firstly, we evaluated the homogeneity of variance using a Levene’s test. For three-group comparisons, we used the Tukey–Kramer test for homogeneous variance; the Steel–Dwass test was used otherwise. For two-group comparisons, Student’s *t* test was used for homogenous variance; the Mann–Whitney *U* test was used otherwise. *P* values <0.05 were considered significant. Statistical analysis was performed using Excel Statistics 2012 (Social Survey Research Information Co., Ltd., Tokyo, Japan) with Excel 2010 (Microsoft Co., Redmond, WA, USA).

## Results

### Metabolite concentrations

The Cramer–Rao lower bound was below 30% for all metabolites except Gln and GABA. Regarding Gln, two asymptomatic carriers and three normal datasets had Cramer–Rao lower bound above 30% and were excluded. Regarding GABA, one asymptomatic carrier had Caramer-Rao lower bound above 30% and was excluded.

**Fig 5.**
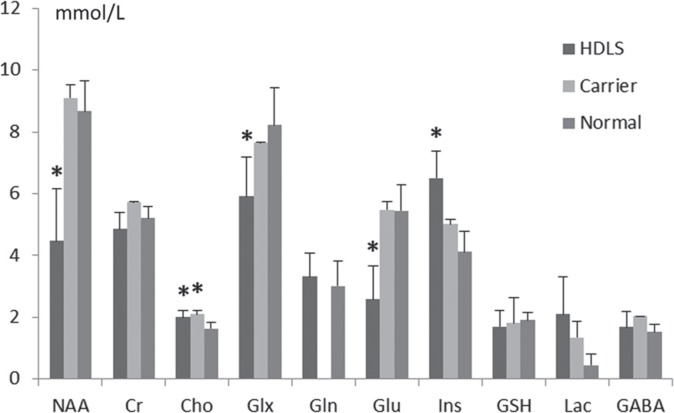
Bar chart showing the mean concentration of each metabolite. Error bars indicate standard deviations. Cho, Ins, and Lac concentrations are increased in patients with HDLS; Cho concentration also increases in asymptomatic carriers, but Ins and Lac concentrations do not increase. NAA, Glx, and Glu concentrations are significantly decreased in patients with HDLS but not in asymptomatic carriers. No significant differences are observed for other metabolites. *, indicates statistical significance (*P* < 0.05). HDLS, Hereditary diffuse leukoencephalopathy with spheroids; NAA, N-acetyl aspartate; Cho, choline-containing compounds; Glx, sum of Gln and Glu; Gln, glutamine; Ins, myo-inositol; Lac, lactate.

Table 2 and Fig. 5 summarize the mean metabolite concentrations. Table 2 also lists each metabolite concentration for patients with HDLS and asymptomatic carriers.

### Statistical analysis

Patient age between the three groups were not significant (*P* value = 0.55 for HDLS vs. normal datasets and 0.93 for asymptomatic carriers vs. normal datasets). Cho and Ins concentrations were increased in patients with HDLS; although Cho was also increased in asymptomatic carriers, Ins was not. NAA, Glx and Glu concentrations were significantly decreased in patients with HDLS; however, these metabolite concentrations were not decreased in asymptomatic carriers. Although no significant differences were observed for other metabolites, a trend was observed for Lac; specifically, Lac was highest in patients with HDLS, followed by asymptomatic carriers, with relatively low, although non-significant, *P* values (0.06 for HDLS vs. normal datasets and 0.09 for asymptomatic carriers vs. normal datasets).

## Discussion

MRS of patients with HDLS revealed a decrease in the NAA concentration, and increase in Cho and Ins concentrations compared to those in normal datasets; these findings were consistent with those of previous studies.^13–15^ We also measured other metabolite concentrations, and observed decreased Glx and Glu concentrations. Cre, Gln, GSH, Lac, and GABA concentrations did not differ significantly between the groups.

To our knowledge, this is the first report to indicate increased Cho concentration in asymptomatic carriers of the causative gene, *CSF1R*. Riku and colleagues reported that the brain of an asymptomatic carrier exhibited pathological changes similar to those observed in patients with HDLS and described those findings as an “early pathologic change”.^16^ Our study therefore contributes new information regarding an “early metabolic change” in the brains of asymptomatic carriers; in other words, brain metabolism is altered even in an asymptomatic state. *CSF1R* is associated with microglia, and pigmented microglia was observed in the brains of patients with HDLS. Increased Cho concentration, which reflects increased cell activity in the brain, could reflect abnormal microglial activity in asymptomatic carriers. Our result was different from previous article;^18^ we proved alterations on metabolites in asymptomatic carriers but a previous article didn’t. There are two possibilities to explain this discrepancy. One is difference of metabolism in asymptomatic carriers; A degree of alterations in metabolism could differ in asymptomatic carriers. Another is previous article might underestimate alterations on metabolites; Figure 4D, an example of MRS of asymptomatic carrier, looks like almost normal; but quantitative analysis proved alterations on metabolites.

As previously reported, brain MRI of asymptomatic carriers may reveal abnormal signal intensity and atrophy, with a gradual extension.^3^ Accordingly, one carrier in the present study exhibited hyperintensity on T_2_WI and atrophy of the frontal and parietal lobes (Fig. 2); however, brain MRI of another carrier did not reveal abnormal findings. In contrast, MRS depicted an increase in Cho levels in both carriers relative to normal datasets, indicating that MRS could likely detect an altered metabolic state before the appearance of MRI abnormalities.

In this study, different patterns of metabolite concentrations were observed in patients with HDLS compared with asymptomatic carriers; NAA, Glx, and Glu concentrations were decreased and Lac was increased in patients relative to normal datasets, whereas this was not true of carriers. The decrease in NAA and increases in Cho and Ins concentrations likely reflect gliosis. A decrease in Glx would probably be caused by a decrease in Glu, an excitatory neurotransmitter; neuronal damage and loss may be a cause of this finding in patients with HDLS.

On the other hand, we observed the same tendency with regard to Ins and Lac concentrations. These were higher in patients with HDLS followed by asymptomatic carriers; however, a significant difference was only observed while comparing Ins levels between patients with HDLS and normal datasets. We assume that gliosis would cause an increase in Ins concentrations. Taken together, our study indicates the presence of altered brain metabolism in asymptomatic carriers, although the degrees of neuronal damage/loss and gliosis are milder than in patients with HDLS. We assumed that the increase of Lac caused by activated microglia; Differentiation of microglia was associated with increase of Lac^20^ and activated microglia was confirmed in previous articles.^2,3,13,16,17^

Accordingly, we can hypothesize the following: 1) changes in metabolites over time could facilitate an understanding of the metabolism of this rare neurodegenerative disease, and 2) MRS could help to screen pedigrees and thus detect asymptomatic members who carry *CSF1R* abnormalities. Further evaluation is needed to prove the usefulness of MRS in these situations.

This study had limitations in addition to its retrospective design. First, this is a rare disease, and as a result, the number of cases was small. For these reasons, we were unable to statistically compare patients with HDLS with asymptomatic carriers. In addition, all patients were female, merely by chance. Furthermore, although Lac exhibited a similar tendency as Ins, the Lac concentrations in patients with HDLS and asymptomatic carriers did not differ significantly from normal datasets. A larger number of cases might reveal statistical differences in other metabolites, including Lac. Second, it can be difficult to quantify Glu, Gln, GSH, and GABA using a 3T clinical scanner. Therefore, we could not determine whether our result in Gln, GSH, and GABA concentrations represented a true or false negative result. However, the quantification of Glx, a combination of Glu and Gln, is a relatively more reliable metabolite analysis. Because Glu and Glx concentrations were decreased in patients with HDLS when compared with normal datasets, Glu may actually be decreased in patients with HDLS.

## Conclusion

We demonstrated changes in metabolite concentrations in patients with HDLS, as well as in asymptomatic carriers of causative gene mutations. Our study indicates that MRS is a potentially useful tool for the analysis of HDLS metabolic and pathophysiological findings, even in the early stages of disease.

## Figures and Tables

**Table 1.** Clinical presentation and MR imaging findings

Clinical presentation						MR imaging findings				
	
ID	Genotype	Clinical status	Age at onset (years)	Age at neuroimaging (years)	Initial symptoms	Atrophy		Hyperintense WM lesions		
	
						Frontoparietal	CC	Frontoparietal		CC

								T_2_WI	DWI	T_2_/DWI
Family 1. III-1	Ala823Val	affected	50	51	cognitive impairment (executive dysfunction)	+	+	confluent PV, deep WM	ovoid, linear deep WM	−
Family 2. III-1	Arg782Gly	affected	33	35	unsteady gait, urinary incontinence, writing difficulty	++	+	confluent subcortical WM	ovoid, polygonal subcortical WM	+
Family 2. II-1	Arg782Gly	asymptomatic		60		±	−	periventricular cap PV WM*	−	−
Family 3. III-1	Arg782Gly	affected	43	45	cognitive impairment (executive dysfunction), writing difficulty	+	+	confluent deep WM	confluentdeep WM	+
Family 3. II-4	Arg782Gly	asymptomatic		67		+	−	confluent PV, deep WM	± deep WM	−
Family 4. III-2	Gly589Arg	affected	48	49	speech problems (difficulties in speaking and finding words)	++**	+	confluent PV, deep WM***	confluent PV, deep WM**	+

*, consistent with age;

**, with diffuse cerebral atrophy;

***, bilateral posterior limb of internal capsule are also involved; WM, white matter; CC, corpus callosum; T_2_WI, T_2_-weighted image; DWI, diffusion-weighted image; PV, periventricular; ±, indicates equivocal finding; MR, magnetic resonance.

**Table 2.** Metabolite concentrations derived from magnetic resonance spectroscopy in patients with HDLS, asymptomatic *CSF1R* mutation carriers, and normal database (mmol/L)

	NAA	Cr	Cho	Glx	Gln	Glu	Ins	GSH	Lac	GABA
HDLS										
Family 1. III-1	6.28	5.46	1.84	7.31	3.53	3.78	5.53	2.29	0.70	2.27
Family 2. III-1	4.16	4.62	1.92	4.78	2.47	2.30	6.79	1.40	2.60	1.28
Family 3. III-1	2.98	4.50	2.26	5.63	3.97	1.67	7.20	1.40	2.98	1.48
mean	4.47*	4.86	2.00*	5.91*	3.32	2.58*	6.51*	1.69	2.09	1.68
*P*-value	<0.01	0.37	0.02	0.02	0.52	<0.01	<0.01	0.64	0.06	0.83
Asymptomatic carrier										
Family 2. II-1	8.78	5.70	2.02	7.65	n.d.	5.31	5.12	2.40	0.96	2.03
Family 3. II-4	9.4	5.75	2.19	7.64	n.d.	5.66	4.91	1.22	1.72	n.d.
mean	9.09	5.73	2.11*	7.65	n.d.	5.49	5.02	1.81	1.34	2.03
*P*-value	0.86	0.20	0.02	0.80	n.d.	1.00	0.22	1.00	0.09	0.46
Normal data										
mean	8.67	5.20	1.64	8.22	3.02	5.45	4.12	1.91	0.45	1.52

HDLS, hereditary diffuse leukoencephalopathy with spheroids; NAA, N-acetyl aspartate; Cr, creatine and phosphocreatine; Cho, choline-containing compounds; Glx, glutamine–glutamate complex; Gln, glutamine; Glu, glutamate; Ins, myo-inositol; GSH, glutathione; Lac, lactate; GABA, gamma-aminobutyric acid; n.d., no data. *P*-values indicate comparisons with normal data;

*, indicates a significant difference.

Statistical analysis, Tukey-Cramer test for NAA, Cr, Cho, Glx, Glu, Ins and GABA, Steel-Dwass test for GSH and Lac, Student’s t test for Gln. HDLS, Hereditary diffuse leukoencephalopathy with spheroids; *CSF1R*, colony-stimulating factor 1 receptor; NAA, N-acetyl aspartate; Cr, creatine and phosphocreatine; Cho, choline-containing compounds; Glx, sum of Gln and Glu; Gln, glutamine; Glu, glutamate; Ins, myo-inositol; GSH, glutathione; Lac, lactate; GABA, gamma-amino butyric acid.
